# Bipolar Transurethral Incision of Bladder Neck Stenoses with Mitomycin C Injection

**DOI:** 10.1155/2015/758536

**Published:** 2015-10-08

**Authors:** Timothy D. Lyon, Omar M. Ayyash, Matthew C. Ferroni, Kevin J. Rycyna, Mang L. Chen

**Affiliations:** Department of Urology, University of Pittsburgh School of Medicine, Pittsburgh, PA 15213, USA

## Abstract

*Introduction*. To determine the efficacy of bipolar transurethral incision with mitomycin C (MMC) injection for the treatment of refractory bladder neck stenosis (BNS). *Materials and Methods*. Patients who underwent bipolar transurethral incision of BNS (TUIBNS) with MMC injection at our institution from 2013 to 2014 were retrospectively reviewed. A total of 2 mg of 40% mitomycin C solution was injected in four quadrants of the treated BNS. Treatment failure was defined as the need for subsequent intervention. *Results*. Thirteen patients underwent 17 bipolar TUIBNS with MMC injection. Twelve (92%) patients had failed a mean of 2.2 ± 1.1 prior endoscopic procedures. Median follow-up was 16.5 months (IQR: 14–18.4 months). Initial success was 62%; five (38%) patients had a recurrence with a median time to recurrence of 7.3 months. Four patients underwent a repeat procedure, 2 (50%) of which failed. Overall success was achieved in 77% (10/13) of patients after a mean of 1.3 ± 0.5 procedures. BNS recurrence was not significantly associated with history of pelvic radiation (33% versus 43%, *p* = 0.9). There were no serious adverse events. *Conclusions*. Bipolar TUIBNS with MMC injection was comparable in efficacy to previously reported techniques and did not result in any serious adverse events.

## 1. Introduction

Bladder neck stenosis (BNS) is a known complication of prostatectomy, prostate radiotherapy, and transurethral resection of the prostate (TURP) [1]. Although the majority of patients can be treated successfully with one to two endoscopic procedures, approximately 27% develop refractory bladder neck stenoses requiring multiple and increasingly complex treatments, potentially culminating in open reconstruction [2–6]. Many go on to require intermittent self-dilation to avoid major reconstruction, which has been shown to decrease quality of life [7]. More effective endoscopic treatments for refractory BNS are therefore needed.

There has been recent enthusiasm for the use of scar modulators such as mitomycin C to help increase success rates of transurethral incision of bladder neck stenoses (TUIBNS). Mitomycin C is a DNA cross-linker that decreases collagen deposition and leads to fibroblast apoptosis [8]. Patency rates for TUIBNS alone in the setting of recurrent stenoses are around 17% per procedure [2]. In contrast, TUIBNS with MMC injection has urethral patency rates ranging from 58 to 72% per procedure [9, 10]; however, MMC injection has also been associated with severe complications including rectourethral fistula, osteitis pubis, and bladder neck necrosis in a minority of patients [9]. Deep cuts into the fat with high dose MMC injection may be the culprit for these complications.

Monopolar electrocautery has greater depth of tissue penetration than bipolar technology [11, 12]. We hypothesize that bipolar cutting current could be associated with increased success following TUIBNS with MMC since less adjacent tissue damage could decrease scar reformation. We also hypothesize that avoiding deep incisions with bipolar electrocautery with MMC could minimize adverse events. To examine these hypotheses, we reviewed our institutional series of bipolar TUIBNS with intralesional MMC injection.

## 2. Materials and Methods

### 2.1. Data Collection

Following institutional review board approval, a retrospective review of all patients who underwent bipolar TUIBNS by a single surgeon (Mang L. Chen) at our institution from January 1, 2013, to December 31, 2014, was completed. Demographic information was collected including age, race, BNS etiology, number and type of prior interventions, and whether or not the patient had previously received pelvic radiation. Operative time and total dose of mitomycin C used were recorded. Postoperative complications were categorized according to the Clavien-Dindo classification [13]. Data were censored as of March 6, 2015.

Postoperatively, patients were monitored every 3 months for stricture recurrence using a combination of serial postvoid residuals, uroflowmetry, and self-reported obstructive voiding symptoms. Flexible cystoscopy was performed as indicated. Recurrence was defined as the need for any subsequent BNS procedure.

### 2.2. Operative Technique

Rigid cystoscopy was performed and guidewire passed through the stenotic lumen into the bladder. Scar resection was accomplished utilizing either a bipolar PK button electrode or PK Plasma-CISE (Gyrus ACMI, Southborough, MA). The PK Plasma-CISE was placed through a traditional 22-French cystoscope; the PK button electrode required use of a 26-French continuous flow resectoscope. The decision as to which instrument to use was made intraoperatively by the attending surgeon on a case-by-case basis. Severe stenosis required cannulation first with the smaller Plasma-CISE. Less severe but symptomatic stenoses were treated first with the Plasma-CISE, and if scar tissue ablation was unsatisfactory for cystoscopic passage into the bladder, the button was used. Scar incision and sometimes resection were accomplished using a cutting current at the 3, 9, and 12 o'clock positions and were continued until the lumen easily permitted the passage of the operating instrument into the bladder. We avoided 6 o'clock incisions to minimize risk of rectal injury. No fat was identified after the incision and/or resection was complete.

Mitomycin C was injected into four quadrants of the treated BNS following incision and/or resection. The needle was advanced approximately 5 mm into the tissue at the 1, 4, 8, and 11 o'clock positions for injection. A total dose of 2 mg of 40% mitomycin C in saline solution was used in all patients. Foley catheter was left in place for 3 days postoperatively.

### 2.3. Data Analysis

Demographic information is reported as frequencies and percentages. Means and standard deviations (SD) are reported for normally distributed data and medians with interquartile ranges (IQR) for nonnormal data. Fisher's exact test was used to compare the likelihood of recurrence between patients who did and did not receive pelvic radiation. Time to recurrence following the initial procedure was modeled with the Kaplan-Meier method. Success rates for initial and repeat procedures were analyzed separately so as not to compare MMC-naïve patients with those who had previously received MMC. Statistical significance was defined at the *p* < 0.05 level using two-tailed tests. Data was analyzed using SPSS software, version 20 (IBM Corp., Armonk, NY).

## 3. Results

Thirteen consecutive patients underwent seventeen bipolar TUIBNS with MMC procedures over the study period. Median follow-up was 16.5 months (IQR: 14–18.4 months). Patient characteristics are summarized in Table 1. Stenosis etiology included radical prostatectomy in 8, prostate brachytherapy in 2, and TURP in 3. Four radical prostatectomy patients received postoperative radiation. Ninety-two percent of patients had failed a mean of 2.2 ± 1.1 prior endoscopic BNS procedures, but all were MMC-naïve.

**Figure 1:**
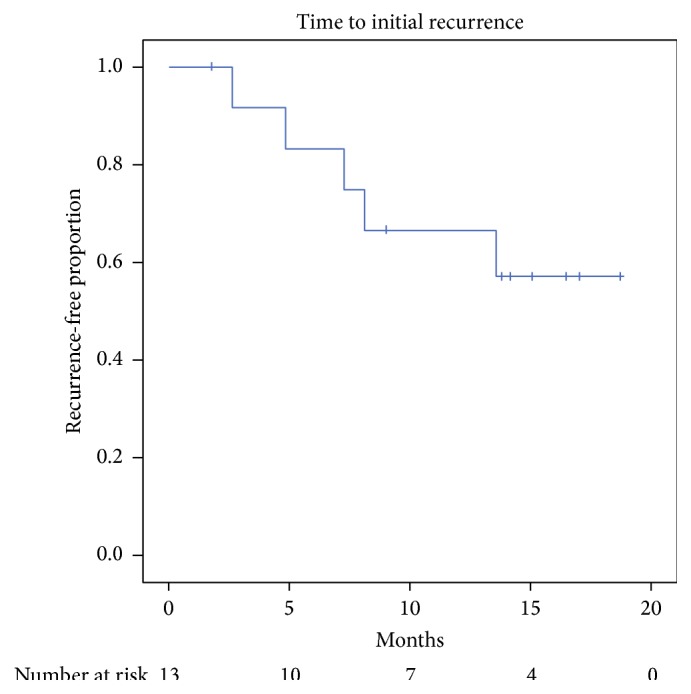
Time to stenosis recurrence following first transurethral incision of bladder neck stenosis with intralesional mitomycin C (MMC) injection in MMC-naïve patients. Censored cases marked by vertical line.

Success following a single bipolar TUIBNS with MMC injection was 62% (8/13), as shown in Figure 1. Five patients (38%) had a recurrence with a median time to recurrence of 7.3 months (IQR: 3.7–10.9 months). Of the five patients who had a recurrence, one was retreated with TUIBNS alone due to a pharmacy shortage and 4 underwent a repeat TUIBNS with MMC procedure. Retreated patients were given the same MMC dosage of 2 mg. Two of the four (50%) repeat MMC procedures failed, one at four months and one at eight months, and these patients did not receive any further MMC with subsequent treatment. Both patients who failed a repeat procedure had a history of pelvic radiation whereas the two patients with successful repeat procedures did not; further the patients who failed repeat procedures had higher Charlson comorbidity index scores (2 and 3 versus 1) than the patients who responded to a second round. Overall, 77% (10/13) of patients had a patent bladder neck after a mean of 1.3 ± 0.5 procedures. Success rate per procedure was 59% (10/17).

Five of thirteen patients (38%) had some degree of stress urinary incontinence prior to undergoing TUIBNS. Incontinence was significantly worsened in two patients (15%) with preexisting incontinence; one subsequently underwent artificial urinary sphincter placement after BNS resolution and the other was managed with a Cunningham clamp per patient preference. Both of these patients had a history of pelvic radiation. De novo incontinence occurred in one patient (8%), which was mild and required one pad/day.

BNS recurrence was not significantly associated with a history of pelvic radiation (33% versus 43%, *p* = 0.9). One postoperative complication occurred, namely, urinary retention after catheter removal necessitating clean intermittent catheterization, classified as Clavien 1.

## 4. Discussion

Management of refractory BNS is a vexing problem for urologists and patients given its high recurrence rates following endoscopic treatments. Approximately 27% of patients with BNS develop refractory stenoses highly resistant to traditional therapy, with success rates of 0–20% following dilation or urethrotomy alone [2, 4]. Injection of mitomycin C as an inhibitor of scar formation has gained some traction for use in this subset of patients. Also, previous work has shown that use of electrocautery over cold knife incision may increase success rates [9]. In this paper we reviewed our institutional series of bipolar TUIBNS with MMC injection to determine whether the addition of bipolar electrocautery could maintain or improve upon previously reported patency rates. Using this approach, we report an initial success rate of 62% (8/13) and an overall patency rate of 77% (10/13) at a median follow-up of 16.5 months, which is consistent with recently reported results [9, 10].

Two previous studies have examined the efficacy of intralesional MMC injection in the treatment of refractory BNS. Vanni and colleagues report a series of 18 patients—all of whom had failed prior endoscopic therapy for BNS—treated with cold knife TUIBNS followed by injection of 0.3–0.4 mg/mL MMC [10]. At a median follow-up of 12 months, they report patency rates of 72% after a single procedure and 89% after two procedures. These are confounding results as other reports have suggested superiority of monopolar TUIBNS over cold knife incision [9, 14]. MMC injection may explain the higher than expected patency rates. Building upon this study, Redshaw et al. (TURNS study) reported a multi-institutional series of 55 patients treated with TUIBNS plus MMC [9]. Eighty percent of included patients had failed prior endoscopic treatment, and mean MMC dose was 3.5 mg. Initial and overall success rates in their series were 58% and 75%, respectively. Patients in the TURNS study had TUIBNS by either cold knife or monopolar incision depending on individual surgeon preference. Interestingly, use of electrocautery was associated with success on univariate analysis (OR 10.7 [95% CI 1.2–197], *p* = 0.03), although the confidence interval was wide and no multivariable risk adjustment was possible due to sample size. Our initial and overall success rates of 62% and 77% compare favorably with these two series. It should be noted, however, that our definition of treatment failure as based on clinical symptoms may be causing us to overestimate success relative to the TURNS experience, which defined recurrence based on urethral caliber noted on cystoscopy regardless of whether obstructive voiding symptoms were present.

Two of four patients in our series who failed an initial TUIBNS with MMC responded to a second round of treatment. Subsequent procedures were performed in an identical manner with the same MMC dosage as initial treatments. Both patients who responded to a second procedure had no history of pelvic radiation and a Charlson comorbidity index of 1; conversely the two patients who failed had both received pelvic radiation and had higher Charlson scores of 2 and 3. Unfortunately comparative statistics were not possible in this subset of patients due to the small overall numbers; however our data suggest that a history of pelvic radiation and greater number of comorbidities may be associated with decreased success rates for subsequent TUIBNS with MMC procedures. This finding needs to be validated in a larger sample size before this statement can be definitively made, however.

It is not clear from the literature that concomitant MMC injection is necessary to achieve high success rates following TUIBNS. In contrast to the above, Ramirez and colleagues were able to demonstrate an overall patency rate of 86% after 2 procedures at a mean follow-up of 13 months in 50 patients who underwent TUIBNS with electrocautery alone [14]. It is also not clear whether our ability to achieve similar success to this is due principally to the use of electrocautery, MMC injection, or a combination of the two. Unfortunately the present study is not designed or powered to delineate these differences.

Data from TURP procedures has shown that bipolar current is associated with a more superficial depth of tissue penetration than monopolar electrocautery, ranging from 0.5–1 mm in bipolar procedures to 3–5 mm in monopolar cases [11, 12]. As such, we hypothesized that bipolar cutting current would be associated with increased success following TUIBNS with MMC as there would theoretically be less adjacent tissue damage and therefore less of an impetus for scar reformation. However, with initial success in 62% of our patients compared to 58% in a series of mixed cold and hot knife procedures [9], we are unable to conclude that bipolar electrocautery meaningfully improved success. Due to our small sample size, the power to detect a true difference between techniques is low, and it is possible that with a larger sample a significant difference between the two techniques could emerge.

No patients in our study required diversion, and the only postoperative complication was one case of retention following catheter removal, requiring clean intermittent catheterization. The TURNS group noted 4 adverse events with 3 patients needing cystectomy [9]. There are two key protocol differences that may account for this discrepancy. First, our study exclusively used a 2 mg dose of mitomycin C, lower than the doses ranging from 0.4 to 10 mg in their series. Second, no perivesicular fat was visualized after incision in our series. This is in stark contrast to nearly every other research protocol where, as a prerequisite to injection, incisions were routinely made until the fat was visualized [9, 10, 14, 15]. These deeper incisions may account for the prevalence of serious complications observed in prior studies by allowing extravasation of MMC into the perivesicular fat, resulting in instances of rectourethral fistula, osteitis pubis, and bladder neck necrosis. It should be noted that our series achieved equivalent urethral patency rates to the TURNS study without requiring deep bladder neck incision into the perivesicular fat.

This study has several significant limitations. As with all retrospective studies, the potential for a selection bias exists. The small number of patients included limits statistical power to find differences in outcome. Follow-up was relatively short with a median of 16.5 months and long-term outcomes in these patients are not known. All patients included in this study were Caucasian, which limits generalizability to those of other racial backgrounds. The makeup of our cohort was heterogeneous, including patients who did and did not receive pelvic radiation as well as those with stenosis developing after varying treatments for both benign and malignant prostatic diseases. Unfortunately, the uncommon nature of this problem is a barrier to reaching a large enough sample size to compare these discrete groups directly, and this is reflected in the sample sizes of 18 and 55 seen in the Vanni and Redshaw papers, respectively [9, 10].

Despite these limitations, our results are meaningful for several reasons. We report the first series to our knowledge using bipolar TUIBNS with concomitant MMC injection for the treatment of refractory BNS. We achieved initial success in 62% and overall success in 77% of patients after a mean of 1.3 procedures, which is comparable to prior studies. Importantly, none of the serious adverse events that have previously been reported with the use of MMC were found in our series, possibly due to the previously described alterations in dosage and technique. Although the sample size is small, our results indicate that TUIBNS with MMC can achieve reasonable success without major morbidity. Further study with larger sample size, less patient heterogeneity, and longer follow-up is needed both to determine whether the use of bipolar electrocautery can confer an advantage over other techniques and to confirm that serious morbidity can effectively be avoided without compromising treatment success.

## 5. Conclusions

Bipolar TUIBNS with MMC done without deep incisions into the perivesicular fat can achieve comparable success to previously published techniques while also avoiding serious adverse events. A prospective, randomized study is needed to determine which factors (dilation, cold knife DVIU, monopolar/bipolar electrocautery, and scar modulator injection) are the most important in decreasing BNS recurrence.

## Figures and Tables

**Table 1:** Patient demographics.

	*n* = 13
Age, years, mean ± SD	67 ± 8.0
BMI, kg/m^2^, mean ± SD	31 ± 6.0
Charlson comorbidity index (%)	
0-1	4 (31)
2	5 (38)
3	4 (31)
Etiology (%)	
RRP	5 (33)
RALP	3 (20)
TURP	3 (20)
Brachytherapy	2 (13)
Prior BNS treatment (%)	12 (92)
Number of prior interventions, mean ± SD	2.2 ± 1.1
Prior radiation (%)	6 (46)

SD: standard deviation, RRP: radical retropubic prostatectomy, RALP: robot-assisted laparoscopic prostatectomy, TURP: transurethral resection of prostate, and TUIBN: transurethral incision bladder neck.
